# Leptin and adiponectin in children and young persons with congenital adrenal hyperplasia

**DOI:** 10.1093/ejendo/lvaf165

**Published:** 2025-08-08

**Authors:** Irina A Bacila, Neil R Lawrence, Sabah Alvi, Timothy D Cheetham, Elizabeth Crowne, Urmi Das, Mehul T Dattani, Justin H Davies, Evelien Gevers, Brian Keevil, Ruth E Krone, Allan Lawrie, Leena Patel, Tabitha Randell, Fiona J Ryan, S Faisal Ahmed, Nils P Krone

**Affiliations:** Division of Clinical Medicine, School of Medicine and Population Health, University of Sheffield, Western Bank, Sheffield S10 2TH, United Kingdom; Division of Clinical Medicine, School of Medicine and Population Health, University of Sheffield, Western Bank, Sheffield S10 2TH, United Kingdom; Department of Paediatric Endocrinology, Leeds General Infirmary, Leeds LS1 3EX, United Kingdom; Great North Children's Hospital, University of Newcastle, Newcastle NE1 7RU, United Kingdom; Bristol Royal Hospital for Children, University Hospitals Bristol Foundation Trust, Bristol BS2 8BJ, United Kingdom; Department of Paediatric Endocrinology, Alder Hey Children's Hospital, Liverpool L14 5AB, United Kingdom; Department of Paediatric Endocrinology, Great Ormond Street Hospital, London WC1N 3BH, United Kingdom; Department of Paediatric Endocrinology, University Hospital Southampton, Southampton SO16 6YD, United Kingdom; Medicine, University of Southampton, Southampton SO17 1BJ, United Kingdom; Centre for Endocrinology, William Harvey Research Institute, Queen Mary University London, London and Barts Health NHS Trust—the Royal London Hospital, London EC1M 6BQ, United Kingdom; Department of Biochemistry, Manchester University NHS Foundation Trust, Manchester M13 9WL, United Kingdom; Department of Paediatric Endocrinology, Birmingham Women's & Children's Hospital, Birmingham B4 6NH, United Kingdom; National Heart and Lung Institute, Faculty of Medicine, Imperial College London, London SW3 6LY, United Kingdom; Department of Paediatric Endocrinology, Royal Manchester Children's Hospital, Manchester University NHS Foundation Trust and Division of Medical Education, the University of Manchester, Manchester M13 9WL, United Kingdom; Department of Paediatric Endocrinology, Nottingham Children's Hospital, Nottingham NG7 2UH, United Kingdom; Oxford Children's Hospital, Oxford University Hospitals NHS Foundation Trust, Oxford OX3 9DU, United Kingdom; Developmental Endocrinology Research Group, University of Glasgow, Glasgow G12 8QQ, United Kingdom; Division of Clinical Medicine, School of Medicine and Population Health, University of Sheffield, Western Bank, Sheffield S10 2TH, United Kingdom

**Keywords:** leptin, adiponectin, congenital adrenal hyperplasia, glucocorticoid treatment, androgens

## Abstract

**Objectives:**

Patients with congenital adrenal hyperplasia (CAH) have increased prevalence of metabolic problems. We studied adiponectin, leptin and resistin in children with CAH, in relation to BMI, treatment, hormonal and metabolic biomarkers.

**Design and Methods:**

We analysed 101 patients with 21-hydroxylase deficiency (54 females, 13.0 ± 2.92 years) from 13 centres in the United Kingdom, and 83 sex- and age-matched controls. Blood parameters (leptin, adiponectin, resistin, metabolic and hormonal markers) were measured in fasted state, between 09:00 and 11:00, after the first glucocorticoid (GC) dose.

**Results:**

A difference in adipokines between patients and controls was only found for leptin in males (patients > control, *P* = .033). In patients and controls, leptin had a positive relationship with BMI-SDS (*P < .001*). However, adiponectin decreased with the BMI only in patients (*P*  *<*  *.001*). Contrary to published evidence on the effect of synthetic steroids on leptin, in our cohort, leptin decreased with the increasing first daily hydrocortisone (HC) dose (*Log_10_Leptin*  *=*  *4.1– 0.08xfirstGCdose (mg/m^2^)*, *P*  *=*  *.009)* but not with the total daily dose. When correcting for BMI, a positive relationship between leptin and insulin was only found in controls (*P*  *<*  *.001*). Adiponectin decreased with steroid precursor and androgen concentrations (17-hydroxyprogesterone, androstenedione, testosterone, 11-hydroxyandrostenedione, 11-ketotestosterone) in patients.

**Conclusion:**

Our findings indicate a decrease in leptin with the HC dose, consistent with a detrimental effect of glucocorticoid on satiety and hunger pathways in CAH. Adiponectin was decreased in patients with increased androgens concentrations, suggesting it may be used as an indicator of metabolic risk associated with poor hormonal CAH control.

Significance Patients with congenital adrenal hyperplasia (CAH) have an increased risk of metabolic disease. In order to help understand the effects of CAH on metabolism during childhood and adolescence, we studied two main adipokines, adiponectin and leptin, in a national cohort of patients aged 8-18 years with CAH. We analysed their relationship with body mass, glucocorticoid treatment and blood biomarkers of disease control. We found that adiponectin decreased with the increase in androgens, including 17-hydroxyprogesterone, androstenedione, testosterone and 11-oxygenated androgens. Leptin decreased with the first hydrocortisone dose of the day. We believe that our work could represent a steppingstone for establishing independent predictors of adverse metabolic outcomes beyond traditional markers of disease control in CAH and other hyperandrogenic states.

## Introduction

Congenital adrenal hyperplasia (CAH) represents a group of inherited conditions characterized by impaired adrenal steroidogenesis. Patients with CAH require life-long treatment with glucocorticoids (GCs). Treatment strategies mimicking the physiological cortisol circadian pattern are difficult to achieve. This is due to the pharmacological properties of synthetic GCs with variable pharmacokinetics among individuals and in the absence of reliable monitoring strategies for disease control.^1,2^ Moreover, while in other forms of adrenal insufficiency the glucocorticoid (GC) treatment aims to merely replace the lacking cortisol, higher doses are required in CAH to normalize the excessive ACTH secretion and hyperandrogenism. Patients with CAH have increased prevalence of metabolic and cardio-vascular comorbidities in both adults^3^ and children^4,5^; however, the mechanisms involved in their development are not clear, and adequate monitoring strategies are lacking.

Glucocorticoids and androgens considerably impact on the regulation of many metabolic pathways.^6-9^ GCs increase blood glucose, by stimulating gluconeogenesis in the liver and decreasing glucose uptake in the adipose tissue and skeletal muscle,^6^ thus antagonising insulin. Moreover, GCs interfere directly with the insulin signalling pathway by directly inhibiting components such as glycogen synthase kinase-3, glycogen synthase, glucose transporter type 4 translocation, further inhibiting insulin-mediated glucose uptake.^10^ GCs induce the activity of lipoprotein lipase, thus increasing the release of fatty acids into the bloodstream, which interferes with glucose utilization and results in insulin resistance.^7^ They promote the differentiation of adipocytes and increase the uptake and turnover of fatty acids in the adipose tissue. Animal and human studies showed that androgens also have a direct effect on lipid metabolism, being regulators of adipocyte differentiation, adiponectin secretion from adipose tissue, lipolysis, lipogenesis and insulin signalling.^8^  ^,9^ Adipokines are also essential regulators of metabolism. Leptin, secreted by the white adipose tissue, acts on hypothalamic nuclei and mediates signalling pathways that regulate food intake and energy expenditure.^11^ However, leptin is also involved in the regulation of glucose homeostasis and insulin signalling.^12^ Adiponectin, an adipokine produced by lean adipose tissue, regulates the synthesis of anti-inflammatory mediators^11^; it is also involved in the metabolic function of many organs and tissues^13^ and has insulin sensitising actions.^14^ Resistin is an adipokine involved in glucose homeostasis, lipid metabolism, and food intake regulation.^15^ Many studies support the role of GCs in modulating central structures involved in food intake and satiety response, as well as their interrelation to adipokines in regulating adipose tissue distribution and function.^16-19^ However, the mechanisms of action and pathological implications of these aspects are not fully understood.^7,20^

In our previously published cohort of 101 children and young persons with CAH caused by 21-hydroxylase deficiency (21OHD),^21^ we found increased frequency of excessive weight in patients, while insulin and HOMA-IR were comparable to healthy controls. Abnormal lipid profiles were present in a small number of patients. Our findings and those of others^4,5^ indicate that metabolic problems in CAH have their onset during childhood or adolescence. This highlights the pressing need for better mechanistic understanding to develop monitoring and prevention strategies. The contribution of adipokines to the metabolic problems associated with CAH is poorly understood. The published evidence is sparse and not fully conclusive, with some studies describing either altered or normal leptin concentrations^22,23^ and others reporting high adiponectin concentrations in children with 21OHD.^24^ In this study, we aimed to explore the relationship of adipokines with body weight, GC treatment and the hormonal biomarkers, as a first step towards gaining more insight into the mechanisms involved in the development of metabolic disease in CAH.

## Methodology

A cross-sectional cohort study was conducted, with the participation of 13 tertiary UK centres, recruiting patients with 21OHD and age- and sex- matched controls (Clinical Trials Registration Number: SCH/15/088).^21^ Data collected consisted of one-time measurement of height, weight, waist and hip circumference, assessment of pubertal development, as well as collection of blood samples. The body surface area was determined using the Mosteller formula.^25^

Blood samples were collected in a fasted state between 09:00 and 11:00 after the first GC dose. The blood samples were analysed for the measurement of biochemical profiles (electrolytes, urea, lipids, glucose, and plasma renin activity) in the respective endocrine tertiary centres; blood steroid hormones (including 17-hydroxyprogesterone, androstenedione, testosterone, 11-hydroxyandrostenedione and 11-ketotestosterone) analysed by liquid chromatography tandem mass spectrometry (LC-MS/MS) at the Biochemistry Department, University Hospital of South Manchester; insulin was measured using enzyme-linked immunosorbent assay (ELISA) at the Clinical Chemistry Department, Sheffield Teaching Hospital Trust; and adipokines were measured by Luminex Multi-Analyte Profiling platform (leptin, resistin) and ELISA (adiponectin) (BioTechne, United States). The presence of insulin resistance was defined by the Homeostatic Model of Insulin Resistance (HOMA-IR) (formula: fasting insulin (µU/L)×glucose (nmol/L)/22.5), using a cut-off 1.68 for normal-weight and 3.42 for overweight individuals based on previously published data from children.^26^

The study was approved by the Yorkshire and Humber Research Ethics Committee and conducted in accordance with the Declaration of Helsinki.

### Statistical analysis

The weight, height and BMI were expressed as standard deviation scores, calculated with the use of the Growth Analyser RCT Software, the chosen reference normative values being the WHO data for the United Kingdom of Great Britain and Northern Ireland. Differences between groups of participants were explored by analysis of variance (Chi-squared, ANOVA, Mann-Whitney U and Fisher’s exact test) and analysis of covariance (ANCOVA or Quade test), with a statistical threshold of significance (*P* value) of < 0.05. We hypothesized on the variables that may have an impact on adipokines, including age, sex, GC dose, time elapsed from dose to sample collection and used correlations and regressions to study these relationships. The statistical work and computation were carried out using R (R Core Team (2021)), SPSS Version 25 and GraphPad Prism 7. For the regression analysis, adipokines and plasma androgen values were log-transformed to reduce the impact of skewed data.

## Results

The demographic characteristics of the CAH-UK cohort and the comparative description of age, sex and pubertal stage distribution between patients and controls were previously reported.^21^ Of the total number of 101 patients (54 females, age 13.0 ± 2.92 years) and 83 age- and sex-matched healthy controls recruited for the CAH-UK cohort, 87 patients and 75 controls were included in the adipokine analysis; the rest were excluded because of missing data (Table 1).

**Table 1.** Demographic characteristics of participants.

	Patients	Controls	Statistical difference
Number	*n* = 87	*n* = 75	
Age (years)	12.5 (10.0-15.)	13.5 (10.7-15.5)	*P* *=* .*273*
Girls	47 (54.0%)	43 (57.3%)	*P* *=* *.492*
Boys	40 (45.9%)	32 (42.6%)	
White	63 (71.6%)	67 (89.3%)	*P < .001*
Asian Pakistani	18 (20.5%)	0	
Asian other	0	3 (4.0%)	
Black ethnicity	1 (1.1%)	2 (2.7%)	
Mixed ethnicity	5 (5.7%)	2 (2.6%)	
Other ethnicities	0	1 (1.3%)	

### The relationship between adipokines and BMI

CAH patients in the present sub-cohort had higher SDS for weight (*P*  *=*  *.003*), BMI (*P*  *<*  *.001*), waist (*P*  *<*  *.001*) and hip circumference (*P*  *=*  *.007*) compared with controls (Table 2). Subgroup analysis demonstrated that it was only in female participants that weight SDS (*P*  *=*  *.022*), BMI SDS (*P*  *=*  *.002*) and waist circumference SDS (*P*  *=*  *.006*) were significantly higher in patients compared with controls, while hip circumference was higher in patients compared with controls only for males (*P*  *=*  *.007*) (Table 2).

**Table 2.** Anthropometric data comparison patients vs controls.

Variable	Patients	Controls	Patients vs Controls
Height SDS			
All	0.4 (−0.8-1.2)	0.1 (−0.4-0.9)	*P* *=* .*54*
	*n* = 87	*n* = 75	
Males	0.5 (−0.6-2.3)	0.1 (−0.5-1.6)	*P* *=* *.282*
	*n* = 40	*n* = 32	
Females	0.1 (−0.8-0.7)	0.1 (−0.4-0.7)	*P* *=* *.843*
	*n* = 47	*n* = 43	
Weight SDS			
All	0.9 (0.1-1.9)	0.2 (−0.4-0.9)	*P* *=* *.003*
	*n* = 87	*n* = 75	
Males	1.2 (0.2-2.1)	0.5 (−0.2-1.7)	*P* *=* *.062*
	*n* = 40	*n* = 32	
Females	0.5 (0.0-1.7)	0.0 (−0.5-0.7)	*P* *=* *.0.22*
	*n* = 47	*n* = 43	
BMI SDS			
All	1.0 (0.0-1.9)	0.2 (−0.3-0.8)	*P* *<* *.001*
	*n* = 87	*n* = 75	*P* *=* *.073*
Males	1.1 (0.0-2.0)	0.4 (−0.2-1.2)	
	*n* = 40	*n* = 32	
Females	0.8 (0.0-1.9)	0.0 (−0.5-1.0)	*P* *=* *.002*
	*n* = 47	*n* = 43	
Waist circumference SDS			
All	1.2 (0.3-2.2)	0.5 (−0.4-1.2)	*P* *<* *.001*
	*n* = 87	*n* = 75	
Males	1.2 (0.3-2.2)	0.6 (−0.3-1.5)	*P* *=* *.070*
	*n* = 40	*n* = 32	
Females	1.3 (0.1-2.3)	0.4 (−0.4-1.0)	*P* *=* *.006*
	*n* = 47	*n* = 43	
Hip circumference SDS			
All	0.9 (−0.6-1.7)	0.0 (−1.1-0.8)	*P* *=* *.007*
	*n* = 87	*n* = 75	
Males	1.3 (0.1-2.3)	0.2 (−1.1-1.3)	*P* *=* *.007*
	*n* = 40	*n* = 32	
Females	0.2 (−1.3-1.5)	−0.1 (−1.1-0.7)	*P* *=* *.304*
	*n* = 47	*n* = 43	

The variables are expressed as median with interquartile ranges of SDS-scores, calculated for age and sex using WHO normative data. The *P* values correspond to the Mann-Whitney U test, for the group and subgroup comparison.

**Figure 1.**
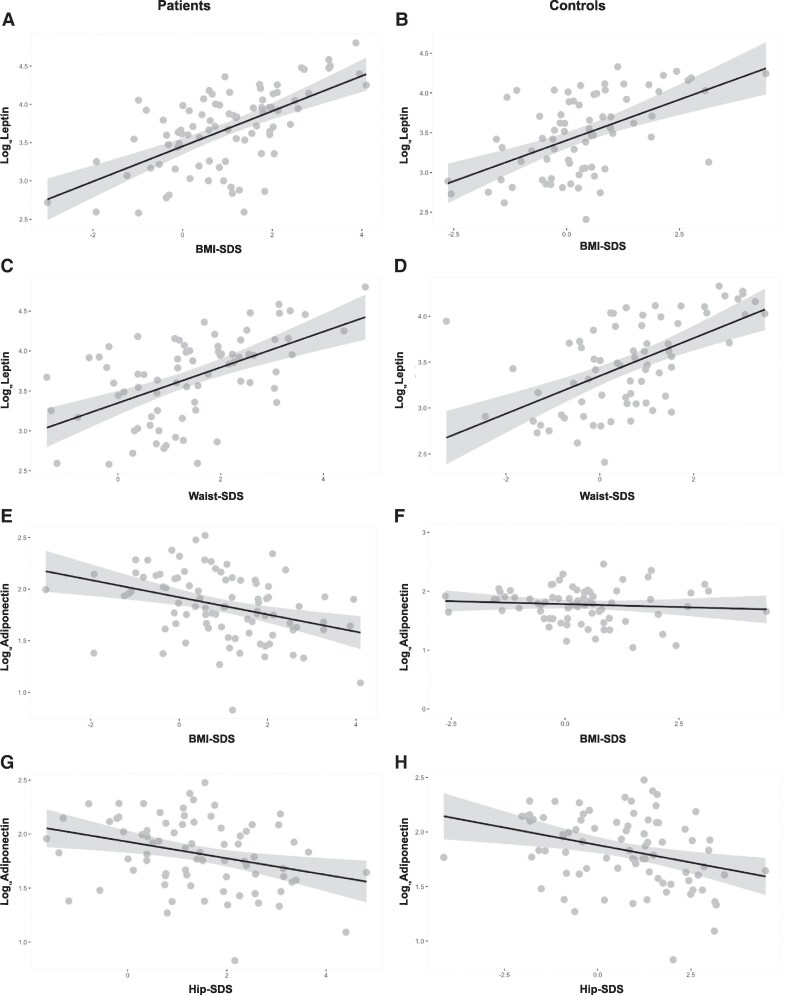
Leptin and adiponectin in relation to BMI, waist and hip SDS. Linear regression showing the relationship between leptin and BMI-SDS in patients (A) and controls (B), leptin and waist-SDS in patients (C) and controls (D), adiponectin and BMI-SDS in patients (E) and controls (F), and adiponectin and hip-SDS in patients (G) and controls (H). The grey ribbons correspond to the 95% confidence interval.

A positive correlation between leptin and BMI-SDS was found in both patients and controls with a similar effect size indicating an increase in Log_10_Leptin of 0.2 with each point of BMI-SDS. (Figure 1A, B and Table 3). There was a positive relationship between leptin and waist-SDS in patients and controls (Figure 1C, D and Table 3). Adiponectin decreased with increasing BMI-SDS in patients; however, the relationship was not significant in controls (Figure 1E, F and Table 3). In CAH patients, adiponectin also had a significant negative relationship with both the waist circumference and the hip circumference SDS (Figure 1G, H and Table 3); this relationship was absent in controls. There was no correlation between resistin and any of the anthropometric measurements in patients or controls.

**Table 3.** Linear regression equations for the relationship of adipokines with BMI, hip and Waist SDS, age, insulin and androgens.

Patients				Controls			
*Log_10_ Leptin*	*= 3.45*	*+ 0*.*22×BMI-SDS*	*P* *<* .*001*	*Log_10_ Leptin*	*= 3*.*40*	*+ 0*.*20×BMI-SDS*	*P* *<* .*001*
*Log_10_ Leptin*	*= 3.34*	*+ 0.22×Waist-SDS*	*P* *<* *.001*	*Log_10_ Leptin*	*= 3.34*	*+ 0.20×Waist-SDS*	*P* *<* *.001*
*Log_10_ Adiponectin*	*= 1.92*	*− 0.08×BMI-SDS*	*P* *<* *.001*	*Log_10_ Adiponectin*	*= 1.720*	*+ 0.02×BMI-SDS*	*P* *=* *.05*
*Log_10_ Adiponectin*	*= 1.92*	*− 0.07×Hip-SDS*	*P* *=* *.001*	*Log_10_ Adiponectin*	*= 1.720*	*− 0.06×Hip-SDS*	*P* *=* *.002*
*Log_10_ LeptinFemales*	*= 3.39*	*+ 0.03×Age*	*P* *=* *.06*	*Log_10_ LeptinFemales*	*= 3.13*	*+ 0.04×Age*	*P* *=* *.04*
*Log_10_ LeptinMales*	*= 5.47*	*− 0.14×Age*	*P* *=* *.08*	*Log_10_ LeptinMales*	*= 4.15*	*− 0.06×Age*	*P* *=* *.32*
*Log_10_ Leptin*	*= 0.58*	*+ 0.15×Log_10_ Insulin*	*P* *=* *.01*	*Log_10_ Leptin*	*= 0.09*	*+ 0.26×Log_10_ Insulin*	*P* *<* *.001*
*Log_10_ Adiponectin*	*= 2.1*	*− 0.15×Log_10_17OHP*	*P* *=* *.003*	*Log_10_ Adiponectin*	*= 1.7*	*− 0.11×Log_10_17OHP*	*P* *=* *.25*
*Log_10_ Adiponectin*	*= 1.9*	*− 0.18×Log_10_A4*	*P* *=* *.006*	*Log_10_ Adiponectin*	*= 1.7*	*− 0.14×Log_10_A4*	*P* *=* *.14*
*Log_10_ Adiponectin*	*= 1.8*	*− 0.17×Log_10_T*	*P* *=* *.007*	*Log_10_ Adiponectin*	*= 1.7*	*− 0.10×Log_10_T*	*P* *=* *.03*
*Log_10_ Adiponectin*	*= 1.9*	*− 0.19×Log_10_11OHA4*	*P* *=* *.008*	*Log_10_ Adiponectin*	*= 1.8*	*− 0.14×Log_10_11OHA4*	*P* *=* *.31*
*Log_10_ Adiponectin*	*= 1.8*	*− 0.16×Log_10_11KT*	*P* *=* *.002*	*Log_10_ Adiponectin*	*= 1.7*	*− 0.16×Log_10_11KT*	*P* *=* *.28*

Hormone abbreviations: 17OHP, 17-hydroxyprogesterone; A4, androstenedione; T, testosterone; 11OHA4, 11-hydroxyandrostenedione; 11KT, 11-ketotestostertone.

### The impact of age and sex on adipokines

**Figure 2.**
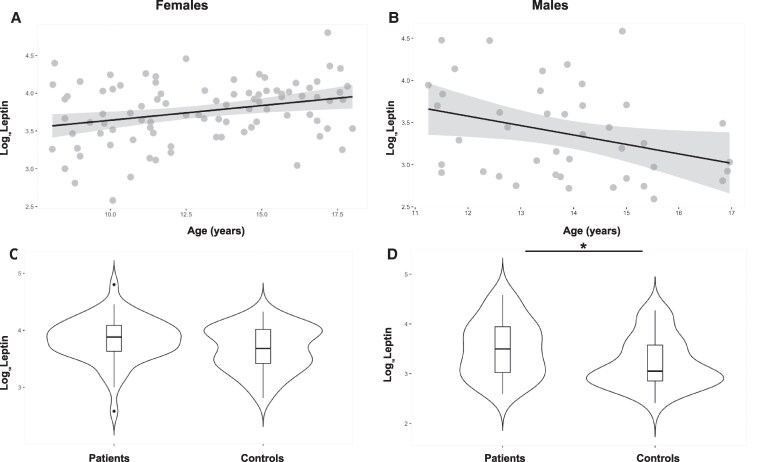
Variations in leptin in relation age and sex. Relationship between leptin in females (all participants) (A) and males aged 11-18 years (B), linear regression showing the relationship between leptin and age; the grey ribbons correspond to the 95% confidence intervals. Difference in leptin between patients and controls in females (C) and males (D), violin plots with median and interquartile range.

The relationship between leptin and age was sex-specific and similar in patients and controls (Table 3). In girls, leptin increased with the age in a linear relationship (linear regression for all female participants: *Log_10_Leptin*  *=*  *3.25*  *+*  *0.03xage (years)*, *P*  *<*  *.001*) (Figure 2A). In boys after puberty, Log Leptin decreased by 0.11 with each year of age, but did not show a relationship in those before puberty or across the whole cohort of boys (linear regression for all pubertal male participants: *Log_10_Leptin*  *=*  *4.9-0.1xage (years)*, *P*  *=*  *.03*) (Figure 2B). Adding BMI-SDS as a covariate was not statistically significant.

Leptin was higher in females than in males in both patients and controls (*P*  *<*  *.01*). The only difference between patients and controls for leptin was found in males, where patients had higher leptin than controls (*P*  *=*  *.028*) (Figure 2C, D). For adiponectin and resistin, there was neither a significant relationship with age nor difference between males and females, or patients and controls.

### Adipokines and GC replacement

**Figure 3.**
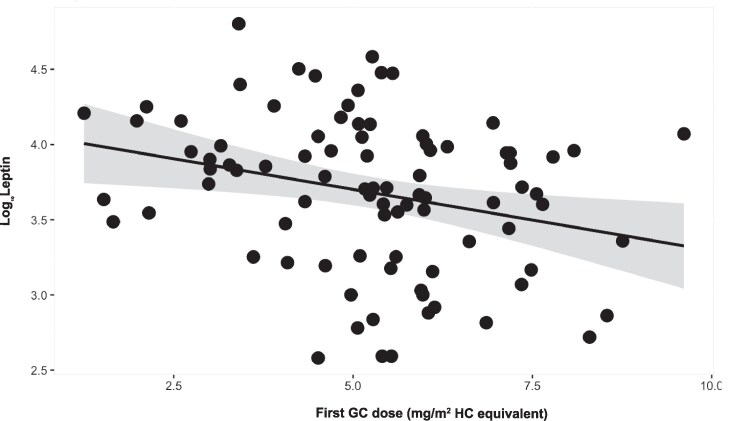
Variations in leptin with the GC dose. Regression analysis of the relationship between leptin and the first daily dose of GC expressed as HC-equivalent in mg/m^2^; the grey ribbon corresponds to the 95% confidence interval.

Bivariate correlations only showed a significant relationship between leptin and the relative first GC dose of the day administered before the blood collection (*r*  *=*  *−0.277, P*  *=*  *.009*), indicating that leptin decreases with the increasing of the GC dose. In patients treated with hydrocortisone (HC) (*n* = 83), regression analysis showed that leptin decreased as the first HC dose of the day increased, *Log_10_Leptin*  *=*  *4.1-0.08xfirstGCdose (mg/m^2^),* R-squared = 0.068, *P*  *=*  *.017* (Figure 3). When converted to the absolute scale, this would equivalate to a decrease in leptin by 843 pg/mL when the first daily HC dose is increased from 5 to 6 mg/m^2^. The time elapsed between the dose administration and the collection of samples varied, with a mean 190 (±152) min. The negative relationship was maintained when correcting for the time elapsed between the dose administration and sample collection. No relationship was found between adiponectin and resistin and the GC dose, or between adipokines and the time of the morning GC dose.

### Adipokines and metabolic markers

**Figure 4.**
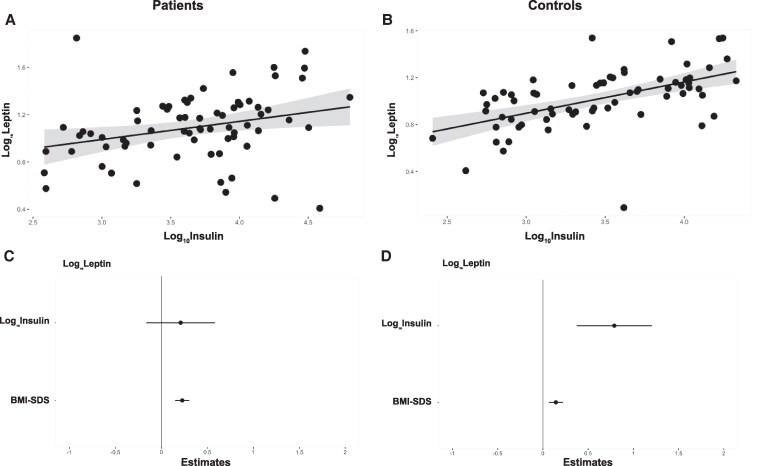
The relationship between leptin and insulin. Regression analysis of the relationship between leptin and insulin in patients (A) and controls (B); the grey ribbons correspond to the 95% confidence intervals. Marginal effect plots of the multivariable regression analysis of leptin with insulin and BMI–SDS in patients (C) and controls (D).

All patients had normal glucose levels. Lipid profiles (LDL, HDL, total cholesterol, triglycerides) were available from 76 patients with CAH. They were normal in the majority of patients; however, there were 4 patients with high cholesterol, 6 with high LDL, 3 with low HDL, and 4 with high triglycerides. Leptin was significantly raised in patients with elevated total cholesterol (*t*-test, *P*  *=*  *.037*) and those with increased LDL (*t*-test, *P*  *=*  *.015*). There was no significant difference between groups defined by lipid profile in the other adipokines. Insulin resistance based on HOMA-IR was found in 53.2% of CAH patients, similar to the prevalence in controls 57.7%. Of the adipokines measured, only leptin was found to have a positive relationship with insulin (and subsequently with HOMA-IR); however, this was much stronger in controls compared with patients (Figure 4A, B and Table 3). When BMI-SDS was added as co-variate, the effect of insulin on leptin remained significant only in controls but not in patients (Figure 4C, D and Table 4).

**Table 4.** Linear regression equations for the relationship between leptin and insulin in both patients and controls.

Patients						Controls					
	DV	Intercept	IV	Covariate	*R* ^2^		DV	Intercept	IV	Covariate	*R* ^2^
	*Log_10_ Leptin=*	*3*.*22*	*+ 0.20 x* *Log_10_ Insulin*	*+ 0.22 x* *BMI-SDS*	*0.39*		*Log_10_ Leptin=*	*2.6*	*+ 0.78 x* *Log_10_ Insulin*	*+0.14 x* *BMI-SDS*	*0.40*
*Coefficient* *P-value*			*P* *=* *0*.*26*	*P* *<* *0.001*	*Model P* *<0.001*	*Coefficient* *P-value*			*P* *<* *0.001*	*P* *<* *0.001*	*Model P* *<.001*

Abbreviations: DV, dependent variable; IV, independent variable.

### Adipokines and plasma androgens

**Figure 5.**
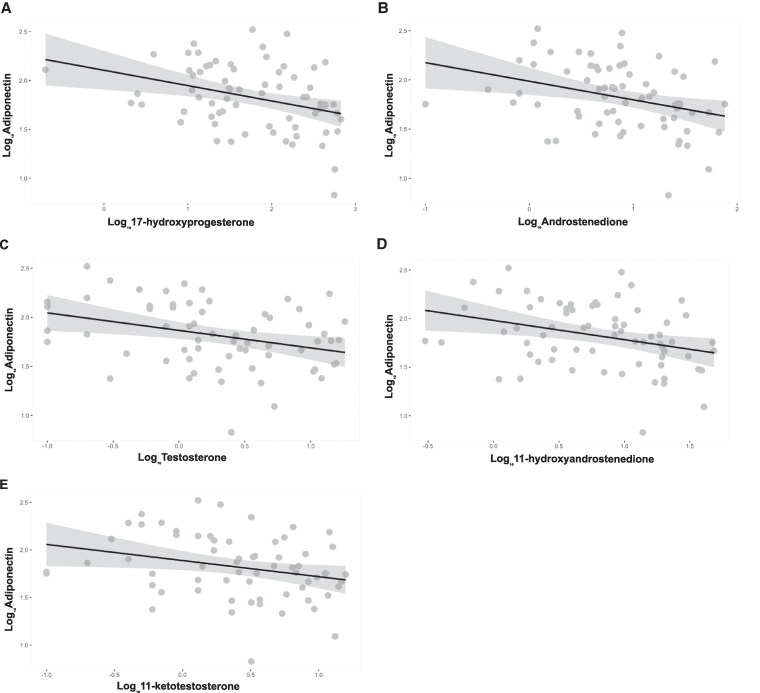
The relationship between adiponectin and plasma biomarkers of hyperandrogenism. Regression analysis of the relationship between adiponectin and 17-hydroxyprogesterone (A), androstenedione (B), testosterone (C), 11-hydroxyandrostenedione (E) and 11-ketotestosterone (F); the grey ribbons correspond to the 95% confidence intervals.

The most significant results in patients were obtained for adiponectin, which had a negative linear relationship with all the measured androgens (Figure 5A-E and Table 3); however, these associations were not found in controls, where adiponectin was only correlated with testosterone. No relationship was found between androgens and BMI-SDS or the GC dose. Resistin did not correlate with any of the measured androgens in patients or controls. For leptin, the only correlation we found was in controls where Log_10_Adiponectin increased by 0.1 with every nmol/L of testosterone (Table 3).

## Discussion

This study aimed to provide a better understanding of the mechanisms involved in the development of metabolic disease in CAH patients during childhood by assessing adipokines. Adipokines are secreted by the adipose tissue and have important roles in processes involved in the regulation of food behaviours and metabolism. The best known and most studied adipokines are leptin and adiponectin, which in this study were found to correlate with the body mass, as well as with the GC dose (leptin), insulin level (leptin) and with plasma androgens in patients with CAH (adiponectin). Resistin was similar between patients and controls and did not have any associations with the anthropometric measurements, or any of the other measured biochemical variables.

Leptin is an established regulator of energy homeostasis, neuroendocrine and metabolic functions.^11,27^ Leptin is secreted by subcutaneous white adipose tissue and activates the leptin receptor in target tissues. In the brain, leptin acts on hypothalamic nuclei and mediates signalling pathways that regulate food intake and energy expenditure.^11^ However, leptin was also found to have direct impact on glucose homeostasis and insulin sensitivity, independent of food intake.^12^ Indeed, it was shown that leptin and insulin influence each other's actions, as both induce the PI3K-AKT pathway involved in insulin signalling.^28^ In previous studies involving CAH patients, leptin correlated positively with fat mass, BMI and cardio-vascular risk in both adults^29^ and children.^22,30^ The raised levels of leptin and insulin found in patients with CAH were attributed to the GC replacement therapy and to the long-term impairment of the adrenal medulla observed in these patients, which may lead to a reduction in the beta-adrenergic suppressive effect on leptin synthesis.^22^ Our results confirmed the correlation between leptin and increased fat mass, both in patients and controls. Of note, in this study we only used BMI, waist and hip circumference as markers for adiposity in CAH, while in general more complex methods, such as bioelectrical impedance or dual-energy X-ray absorptiometry, are recommended for increased precision.^31^ This needs to be taken into consideration, as patient with CAH may have increased muscle mass due to hyperandrogenism. Regarding the difference between patients and controls, in our cohort leptin was higher only in male patients, indicating that the effects of CAH on adipose tissue may be more complex that previously described. It is important to note that there was a significant difference in leptin between males and females in both patients and controls. This difference was reported before with females having higher leptin levels.^32^ Possible explanations offered for this difference included variations in hypothalamic regulation, leptin sensitivity, sex hormones profiles and fat disposition patterns. The latter theory is supported by evidence showing that subcutaneous fat secretes more leptin compared with visceral fat.^33,34^ In our cohort, only male patients had higher hip circumference SDS, suggesting increased subcutaneous fat mass, compared with controls,^21^ which may explain the significant difference in leptin between male patients and male controls.

This study is the first to show a relationship between leptin and age in children. Interestingly, we found that the variations of leptin with age were also sex-specific, as leptin increased with age in females and decreased with it in pubertal males. To our knowledge this is the first study reporting the relationship of leptin with age in children, while data was inconclusive in human adults.^35^

Exploring the effects of CAH medication on adipokines, we found the reduction of leptin levels with the increase of the first relative GC dose of the day. However, we did not observe this with the total daily dose. Our findings are not in keeping with previously published evidence, wherein patients with obesity, dexamethasone treatment increased leptin levels.^33,36^ Administration of dexamethasone in murine models was found to decrease lipopolysaccharide induced leptin secretion,^37^ however, it caused an increase in plasma leptin in adrenalectomized rats^38^ and in obese rats.^39^ There is also evidence showing a difference in the magnitude of the effect on leptin of dexamethasone and prednisolone in patients with leukaemia, although both of them increased the leptin to BMI ratio over a number of weeks.^40^ Notably, dexamethasone is very rarely used in children with CAH and most patients in our study were treated with HC (*n* = 83). Moreover, the leptin measurement was conducted on samples collected within 3 h from the morning HC dose, thus our findings related to the immediate effect of HC on leptin. Our results would suggest an immediate effect of decreased leptin response following the administration of HC; it could be speculated that this may explain the increased appetite reported by patients treated with GC; however, further work is required to test this theory. Other confounding variables, such as the timing of the samples or the plasma androgen concentrations should be considered; however, the statistical analysis did not support their effect. Based on the cross-talk existent between circadian rhythm, cortisol and leptin signalling, it is possible that the relationship we found between the first HC dose of the day and leptin may be related to the modulatory effect that GCs have on leptin secretion.^41^ Thus, this finding highlights the need for further research exploring circadian fluctuations in the leptin secretion in relation with the hormone replacement doses, for better understanding of the impact that GCs have on metabolism and energy homeostasis.

We found that the relationship between leptin and insulin was different between patients and controls. When corrected for BMI, it was only in controls that leptin increased with insulin, but not in CAH patients. Insulin and leptin are known to have a bidirectional relationship where they influence one another in the regulation of glucose homeostasis and appetite,^12,28^ and our results from the control group are also supporting the relationship between them. Our results indicate that this relationship is impaired in patients. We believe that this could be in part a consequence of the GC treatment, which may interfere with the relationship between leptin and insulin, as GCs influence the insulin signalling pathway.^42^ However, other potential factors should also be considered, including hyperandrogenism and insulin or leptin resistance.

The negative relationship between leptin and testosterone was described by a previous study that associated raised leptin with good androgen control in CAH.^43^ However, in our analysis this association was only found in male patients and there was no relationship found between leptin and the other markers of hyperandrogenism, including 17-hydroxyprogesterone and androstenedione which are commonly used in clinical practice, as well as the two 11-oxygenated androgens, 11-hydroxyandrostenedione and 11-ketotestosterone.

Adiponectin has been described as one of the adipokines characteristic to lean adipose tissue, where it induces the production of anti-inflammatory mediators such as interleukins 1 and 10.^11^ It is known to regulate the metabolic function of many organs and tissues^13^ and have insulin sensitising actions.^14^ Reduced adiponectin levels are associated with increased fat mass and insulin resistance.^14^ In patients with CAH adiponectin was reported to be increased in both children^23^ and adults.^4^ By contrast, our patients and controls had comparable adiponectin levels, despite the fact that patients had a higher prevalence of obesity and overweight, as previously reported.^21^ Adiponectin had a negative linear relationship with BMI in our CAH cohort, which is consistent with previously published evidence^23^; however, this was only statistically significant in patients, not in controls. Since the variability in BMI was significant in the control group (between −2.6 and +4.4 SDS), we would have expected a more marked impact on adiponectin and we do not have a clear explanation for this finding in our cohort.

An important finding was the relationship between adiponectin and the measured plasma androgens and androgen precursors in patients with CAH. We believe that the absence of these correlations in controls was likely related to the significantly lower concentrations of androgens in this group.^44^ The studies discussing the relationship between adiponectin and androgens in CAH are limited to a similar size cohort showing correlation between adiponectin and testosterone and dehydroepiandrosterone sulphate^24^ and a more recent but smaller (*n* = 13) cohort that reported a negative relationship with androstenedione.^45^ Our study demonstrates a consistent decrease in adiponectin in patients with higher routine biomarkers (17-hydroxyprogesterone, androstenedione and testosterone) and adrenal specific 11-oxygenated androgens (11-hydroxyandrostenedione and 11-ketotestosterone). Of note, androgens were reported to have a direct effect in the regulation of adiponectin secretion from adipose tissue,^8^ however, the study of their relationship in CAH is relatively limited. The novelty of the current study lies in establishing an association between adiponectin, which is a marker of metabolic risk and an expanded steroid profile of androgen control, including two 11-oxygenated androgens, which are currently explored as potential superior markers of disease control in CAH.^44,46^ This suggests that adiponectin warrants further investigation through long-term follow-up studies as a predictor of metabolic problems in relation to androgen control in patients with CAH.

In summary, our study provides valuable insight regarding the relationship of two major adipokines, leptin and adiponectin, with BMI, GC dose and plasma androgens in children with CAH. Adipokines and cytokines have complex roles that link the hormonal functions of adipose tissue with inflammation, with a wide impact on the development of metabolic and cardiovascular disease. Our results indicated that leptin decreased with the morning HC dose, and we suspect that the development of steroid-induced insulin resistance also involves an impaired signalling dialogue between leptin and insulin. Moreover, adiponectin was decreased in patients with increased androgens, suggesting it may be used an indicator of metabolic risk associated with poor disease control. Longer term follow-up is needed, as well as establishing reliable outcome measures to provide robust information on the risk of developing metabolic disease in CAH and the mechanisms involved. This will help to establish independent predictors of adverse metabolic outcomes beyond traditional markers of disease control in CAH and other hyperandrogenic states.

## Data Availability

The datasets generated and analysed in this study are not publicly available but are available from the corresponding author on reasonable request.
